# Autoimmune disease and COVID-19- a multicentre observational study in the United Kingdom

**DOI:** 10.1093/rheumatology/keac209

**Published:** 2022-04-04

**Authors:** Deepa J Arachchillage, Indika Rajakaruna, Charis Pericleous, Philip L R Nicolson, Mike Makris, Mike Laffan, the CA-COVID-19 study group, Amanda Dell, Angela Hall, Anna Roynon, Anne Heron, Cheri Price, Claire Price, Clare Westacott, Debra Barnett, Gail Marshall, Gemma Hodkinson, Georgia Mallison, Grace Okoro, Joshua Gwatkin, Kirstin Davies, Lucy Shipp, Maxine Nash, Rhian Hughes, Rina Mardania, Sarah Lewis Sean Cutler, Caroline Allan, Atiqa Miah, Dide Okaygun, Dan Hart, Faith Dzumbunu, James Leveson, Karen Torre, Louise Taylor, Priyanka Raheja, Sara Mamsa, Tasnima Ferdousi, Angharad Everden, Alice Ngumo, Doaa Ahmed, Efstathia Venizelou, James Herdman, Janice Carpenter, Konrad Bartkiewicz, Rebecca Cash, Renu Riat, Abigail Downing, Ana Guerrero, Astrid Etherington, Chapa Gamage, Dilupa Gunasekara, Lee Morris, Raza Alikhan, Rebecca Cloudsdale, Samya Obaji, Stuart Cunningham, Sylvain Ndjombo, Amanda Cowton, Ami Wilkinson, Andrea Kay, Anne Sebakungu, Anne Thomson, Clare Brady, Dawn Egginton, Ellen Brown, Enid Wright, Gill Rogers, Hannah Plaschkes, Jacqui Jennings, Julie O’Brien, Julie Temple, Kathryn Potts, Kimberly Stamp, Kelly Postlethwaite, Louise Duncan, Margaret Randall, Mark Birt, Melanie Kent, Philip Mounter, Shelly Wood, Nicola Hewitson, Noreen Kingston, Susan Wadd, Sarah McAuliffe, Stefanie Hobson, Susan Riley, Suzanne Naylor, Vicki Atkinson, Alysha Hancock, Bethan Deacon, Carla Pothecary, Caroline Hamilton, Ceri Lynch, Cerys Evenden, Deborah Jones, Ellie Davies, Felicity Page, Gareth Kennard-Holden, Gavin John, Joanne Pugh, Joelle Pike, Justyna Mikusek, Kevin Agravante, Kia Hancock, Lauren Geen, Meryl Rees, Natalie Stroud, Amanda Grahamslaw, Amanda Sanderson, Beverley McClelland, Caitlin Barry, Elaine Siddle, Lorraine Pearce, Lucy Blackwell, Maria Bokhari, Maureen Armstrong, Wendy Stoker, Wendy McCormick, Caterina Vlachou, Ben Garfield, Mihaela Gaspar, Maurizio Passariello, Paolo Bianchi, Stephane Ledot, Aileen Madlin, Kerrianne Everard, Khushboo Panwar, Natasha Beacher, Niamh Cole, Sarah Mangles, Tamara Everington, Udaya Reddy, Alka Shah, Anna Weatherill, Anthi Maropoulou, Bhagya Herath, Billy Hopkins, Camelia Vladescu, Caroline Ward, Christina Crossette-Thambiah, Donna Copeland, Emily Pickford, Gaurika Kapoor, Isabella Lo, John Kilner, Keith Boland, Melanie Almonte, Neil Simpson, Niamh Bohnacker, Omolade Awomolo, Roochi Trikha, Samina Hussain, Serah Duro, Sophie Kathirgamanathan, Yasmine Needham, Yee Hui, Zainab Alashe, Adrienne Abioye, Aileen Miranda, Christina Obiorah, Cynthia Dzienyo, Hasina Mangal, Hernan Zorraquino, Lara N Roberts, Mariusz Racz, Maclaine Hipolito Johnson, Rachel Ryan, Tamara Swales, Tatiana Taran, Zoe Renshaw, Alexander Langridge, Benjamin Evans, Callum Weller, Claire Judd, Douglas Jerry, Euan Haynes, Fatima Jamil, Ian McVittie, John Hanley, Julie Parker, Kayleigh Smith, Keir Pickard, Laura Kennedy, Meghan Acres, Mikaela Wiltshire, Nitha Ramachandran, Paul McAlinden, Paula Glancy, Smeera Nair, Tarek Almugassabi, Thomas Jarvis, Amanda Coutts, Andrew Laurie, Deborah Owen, Ian Scott, Jamie Cooper, Leia Kane, Lucy Sim, Mahmoud Abdelrahman, Victoria Poulton, Jessica Griffin, Ria Markwell, Suzanne Docherty, Alexander Brown, Barbara Cooper, Beverley Wilkinson, Diane Armstrong, Grace Fryer, Jane Gregory, Katherine Davidson, Melanie Clapham, Nicci Kelsall, Patricia Nicholls, Rachel Hardy, Roderick Oakes, Rosemary Harper, Sara Abdelhamid, Theresa Cooper, Una Poultney, Zoe Saunders, Alex Ramshaw, Alison Chilvers, Barbara Jean Campbell, Carol Adams, Claire Riley, Deborah Wilson, Helen Wardle, Jill Deane, Jill Skelton, Julie Quigley, Leigh Pollard, Liz Baker, Lynda Poole, Maria Weetman, Michele Clark, Nini Aung, Rachel Taylor, Sarah Rowling, Sarah Purvis, Vicky Collins, Amy Shenfine, Catherine Ashbrook-Raby, Charlotte Bomken, Claire Walker, Faye Cartner, Helen Campbell, Jane Luke, Jessica Reynolds, Mari Kilner, Laura Winder, Linda Patterson, Lisa Gallagher, Nicola McLarty, Sandra Robinson, Steve Dodds, Toni Hall, Victoria Wright, Agnes Eordogh, Alexandros Rampotas, Anna Maria Sanigorska, Christopher Deane, Kristine Santos, Olivia Lecocq, Rochelle Lay, Simon Fletcher, Susie Shapiro, Anna Tarnakina, Aniqa Tasnim, Anja Drebes, Cecilia Garcia, Elsa Aradom, Mariarita Peralta, Michaella Tomlin, Pratima Chowdary, Ramona Georgescu, Suluma Mohamed, Upuli Dissanayake, Carol Powell, James Doolan, Jessica Kenworthy, Joanne Bell, Lewis Jones, Mikiko Wilkinson, Rebecca Shaw, Ryan Robinson, Saman Mukhtar, Shane D'Souza, Tina Dutt, Tracy Stocks, Joshua Wade, Lenka Cagova, Maksym Kovzel, Rachel Jooste, Alison Delaney, Claire Mapplebeck, Alycon Walker, Andrea Watson, Andrew Vaux, Asia Sawar, Carol Hannaway, Charlotte Jacobs, Claire Elliot, Claire Elliott, Craig Mower, Daiana Ferro, Emanuela Mahmoud, Gill Laidlaw, Julie Potts, Keith Harland, Laura Munglani, Lauren Fall, Leanne Murray, Lesley Harris, Lisa Wayman, Lisa Westwood, Louisa Watson, Lynne Naylor, Matthew Siddaway, Paula Robson, Rita Mohan, Sarah Essex, Sara Griffiths, Steven Liggett, Andreia Valente, Rashid Kazmi, Ruth Kirby, Sarah Bowmer, Yanli Li, Alice Longe, Amy Bamford, Anand Lokare, Andrew McDarby, Aneta Drozd, Cathy Stretton, Catia Mulvihill, Charlotte Ferris, Christopher McGhee, Claire McNeill, Colin Bergin, Daniella Lynch, Fionnuala Lenehan, Gerry Gilleran, Gillian Lowe, Graham McIlroy, Helen Jenner, Helen Shackleford, Isma Younis, Jaspret Gill, Jimmy Musngi, Joanne Dasgin, Joanne Gresty, Joseph Nyaboko, Juneka Begum, Katerine Festejo, Katherine Lucas, Katie Price, Khushpreet Bhandal, Kristina Gallagher, Kyriaki Tsakiridou, Lauren Cooper, Louise Wood, Lulu Amutike, Marie Thomas, Marwan Kwok, Melanie Kelly, Michelle Bates, Nafeesah Ahmad Haider, Nicholas Adams, Oliver Topping, Rachel Smith, Rani Maria Joseph, Salma Kadiri, Samantha Caddick, Samuel Harrison, Shereef Elmoamly, Stavroula Chante, Sumaiyyah Gauhar, Syed Ashraf, Tabinda Kharodia, Zhane Peterkin, Isgro Graziella, Hakeem Yusuff, David Sutton, Ian Massey, Jade Di-Silvestro, Joanne Hiden, Mia Johnson, Richard Buka, Claire Lentaigne, Jackie Wooding, Nicola Crosbie, Ana Alvaro, Emma Drasar, Elen Roblin, Georgina Santiapillai, Kathryn Simpson, Kayleigh Gilbert, Yanrong Jiang, Zara Sayar, Zehraa Al-Khafaji

**Affiliations:** Centre for haematology, Department of Immunology and Inflammation, Imperial College London, London, UK; Department of haematology, Imperial College Healthcare NHS Trust, London, UK; University of East London, Department of Computer Science, London, University Way, London, E16 2RD, UK; National Heart and Lung Institute, Imperial College London, London, UK; University of Birmingham, Institute of Cardiovascular Sciences, Edgbaston, Birmingham, B15 2TT, UK; Sheffield Teaching Hospitals NHS Foundation Trust, Department of Haematology, Royal Hallamshire Hospital, Glossop Rd, Broomhall, Sheffield, S10 2JF, UK; Centre for haematology, Department of Immunology and Inflammation, Imperial College London, London, UK; Department of haematology, Imperial College Healthcare NHS Trust, London, UK

**Keywords:** Autoimmune rheumatologic disease, COVID-19, mortality, thrombosis, bleeding, antiphospholipid syndrome, Systemic lupus erythematosus, Rheumatoid arthritis

## Abstract

**Objective:**

To establish the demographic characteristics, laboratory findings and clinical outcomes in patients with autoimmune disease (AD) in comparison to a propensity matched cohort of patients without AD admitted with COVID-19 to hospitals in the UK.

**Methods:**

This is a multicentre observational study across 26 NHS Trusts. Data were collected both retrospectively and prospectively using a pre-designed standardised case record form. Adult patients (≥18 years) admitted between 1st of April 2020 and 31 July 2020 were included.

**Results:**

Overall, 6288 patients were included to the study. Of these, 394 patients had AD prior to admission with COVID-19. Of 394 patients, 80 patients with systemic lupus erythematosus, rheumatoid arthritis or antiphospholipid syndrome were classified as severe rheumatologic AD. A higher proportion of those with AD had anaemia: 240(60.91%) *vs* 206(52.28%), *p*= 0.015, raised LDH 150(38.08%) *vs* 43(10.92%), *p*< 0.001 and raised creatinine 122(30.96%) *vs* 86(21.83%), *p*= 0.01 respectively. A significantly higher proportion of patients with severe rheumatologic AD had raised CRP : 77(96.25%) *vs* 70(87.5%), *p*= 0.044 and LDH 20(25%) *vs* 6(7.5%), *p*= 0.021. Patients with severe rheumatologic AD had significantly higher mortality [32/80(40%)] compared with propensity matched cohort of patients without AD [20/80(25%)], *p*= 0.043. However, there was no difference in 180-day mortality between propensity matched cohorts of patients with or without AD in general, *p*= 0.47.

**Conclusions:**

Patients with severe rheumatologic AD had significantly higher mortality. Anaemia, renal impairment and raised LDH were more frequent in patients with any AD whilst raised CRP and LDH were more frequent in patients with severe rheumatologic AD both of which have been shown to associate with increased mortality in patients with COVID-19.

## Supplementary Material

keac209_Supplementary_Data

