# Autoimmune disease and COVID-19: a multicentre observational study in the United Kingdom

**DOI:** 10.1093/rheumatology/keac209

**Published:** 2022-04-04

**Authors:** Deepa J Arachchillage, Indika Rajakaruna, Charis Pericleous, Philip L R Nicolson, Mike Makris, Mike Laffan, the CA-COVID-19 Study Group, Amanda Dell, Angela Hall, Anna Roynon, Anne Heron, Cheri Price, Claire Price, Clare Westacott, Debra Barnett, Gail Marshall, Gemma Hodkinson, Georgia Mallison, Grace Okoro, Joshua Gwatkin, Kirstin Davies, Lucy Shipp, Maxine Nash, Rhian Hughes, Rina Mardania, Sarah Lewis Sean Cutler, Caroline Allan, Atiqa Miah, Dide Okaygun, Dan Hart, Faith Dzumbunu, James Leveson, Karen Torre, Louise Taylor, Priyanka Raheja, Sara Mamsa, Tasnima Ferdousi, Angharad Everden, Alice Ngumo, Doaa Ahmed, Efstathia Venizelou, James Herdman, Janice Carpenter, Konrad Bartkiewicz, Rebecca Cash, Renu Riat, Abigail Downing, Ana Guerrero, Astrid Etherington, Chapa Gamage, Dilupa Gunasekara, Lee Morris, Raza Alikhan, Rebecca Cloudsdale, Samya Obaji, Stuart Cunningham, Sylvain Ndjombo, Amanda Cowton, Ami Wilkinson, Andrea Kay, Anne Sebakungu, Anne Thomson, Clare Brady, Dawn Egginton, Ellen Brown, Enid Wright, Gill Rogers, Hannah Plaschkes, Jacqui Jennings, Julie O'Brien, Julie Temple, Kathryn Potts, Kimberly Stamp, Kelly Postlethwaite, Louise Duncan, Margaret Randall, Mark Birt, Melanie Kent, Philip Mounter, Shelly Wood, Nicola Hewitson, Noreen Kingston, Susan Wadd, Sarah McAuliffe, Stefanie Hobson, Susan Riley, Suzanne Naylor, Vicki Atkinson, Alysha Hancock, Bethan Deacon, Carla Pothecary, Caroline Hamilton, Ceri Lynch, Cerys Evenden, Deborah Jones, Ellie Davies, Felicity Page, Gareth Kennard-Holden, Gavin John, Joanne Pugh, Joelle Pike, Justyna Mikusek, Kevin Agravante, Kia Hancock, Lauren Geen, Meryl Rees, Natalie Stroud, Amanda Grahamslaw, Amanda Sanderson, Beverley McClelland, Caitlin Barry, Elaine Siddle, Lorraine Pearce, Lucy Blackwell, Maria Bokhari, Maureen Armstrong, Wendy Stoker, Wendy McCormick, Caterina Vlachou, Ben Garfield, Mihaela Gaspar, Maurizio Passariello, Paolo Bianchi, Stephane Ledot, Aileen Madlin, Kerrianne Everard, Khushboo Panwar, Natasha Beacher, Niamh Cole, Sarah Mangles, Tamara Everington, Udaya Reddy, Alka Shah, Anna Weatherill, Anthi Maropoulou, Bhagya Herath, Billy Hopkins, Camelia Vladescu, Caroline Ward, Christina Crossette-Thambiah Donna Copeland, Emily Pickford, Gaurika Kapoor, Isabella Lo, John Kilner, Keith Boland, Melanie Almonte, Neil Simpson, Niamh Bohnacker, Omolade Awomolo, Roochi Trikha, Samina Hussain, Serah Duro, Sophie Kathirgamanathan, Yasmine Needham, Yee Hui, Zainab Alashe, Adrienne Abioye, Aileen Miranda, Christina Obiorah, Cynthia Dzienyo, Hasina Mangal, Hernan Zorraquino, Lara N Roberts, Mariusz Racz, Maclaine Hipolito Johnson, Rachel Ryan, Tamara Swales, Tatiana Taran, Zoe Renshaw, Alexander Langridge, Benjamin Evans, Callum Weller, Claire Judd, Douglas Jerry, Euan Haynes, Fatima Jamil, Ian McVittie, John Hanley, Julie Parker, Kayleigh Smith, Keir Pickard, Laura Kennedy, Meghan Acres, Mikaela Wiltshire, Nitha Ramachandran, Paul McAlinden, Paula Glancy, Smeera Nair, Tarek Almugassabi, Thomas Jarvis, Amanda Coutts, Andrew Laurie, Deborah Owen, Ian Scott, Jamie Cooper, Leia Kane, Lucy Sim, Mahmoud Abdelrahman, Victoria Poulton, Jessica Griffin, Ria Markwell, Suzanne Docherty, Alexander Brown, Barbara Cooper, Beverley Wilkinson, Diane Armstrong, Grace Fryer, Jane Gregory, Katherine Davidson, Melanie Clapham, Nicci Kelsall, Patricia Nicholls, Rachel Hardy, Roderick Oakes, Rosemary Harper, Sara Abdelhamid, Theresa Cooper, Una Poultney, Zoe Saunders, Alex Ramshaw, Alison Chilvers, Barbara Jean Campbell, Carol Adams, Claire Riley, Deborah Wilson, Helen Wardle, Jill Deane, Jill Skelton, Julie Quigley, Leigh Pollard, Liz Baker, Lynda Poole, Maria Weetman, Michele Clark, Nini Aung, Rachel Taylor, Sarah Rowling, Sarah Purvis, Vicky Collins, Amy Shenfine, Catherine Ashbrook-Raby, Charlotte Bomken, Claire Walker, Faye Cartner, Helen Campbell, Jane Luke, Jessica Reynolds, Mari Kilner, Laura Winder, Linda Patterson, Lisa Gallagher, Nicola McLarty, Sandra Robinson, Steve Dodds, Toni Hall, Victoria Wright, Agnes Eordogh, Alexandros Rampotas, Anna Maria Sanigorska, Christopher Deane, Kristine Santos, Olivia Lecocq, Rochelle Lay, Simon Fletcher, Anna Tarnakina, Aniqa Tasnim, Anja Drebes, Cecilia Garcia, Elsa Aradom, Mariarita Peralta, Michaella Tomlin, Pratima Chowdary, Ramona Georgescu, Suluma Mohamed, Upuli Dissanayake, Carol Powell, James Doolan, Jessica Kenworthy, Joanne Bell, Lewis Jones, Mikiko Wilkinson, Rebecca Shaw, Ryan Robinson, Saman Mukhtar, Shane D'Souza, Tina Dutt, Tracy Stocks, Joshua Wade, Lenka Cagova, Maksym Kovzel, Rachel Jooste, Alison Delaney, Claire Mapplebeck, Alycon Walker, Andrea Watson, Andrew Vaux, Asia Sawar, Carol Hannaway, Charlotte Jacobs, Claire Elliot, Claire Elliott, Craig Mower, Daiana Ferro, Emanuela Mahmoud, Gill Laidlaw, Julie Potts, Keith Harland, Laura Munglani, Lauren Fall, Leanne Murray, Lesley Harris, Lisa Wayman, Lisa Westwood, Louisa Watson, Lynne Naylor, Matthew Siddaway, Paula Robson, Rita Mohan, Sarah Essex, Sara Griffiths, Steven Liggett, Andreia Valente, Rashid Kazmi, Ruth Kirby, Sarah Bowmer, Yanli Li, Alice Longe, Amy Bamford, Anand Lokare, Andrew McDarby, Aneta Drozd, Cathy Stretton, Catia Mulvihill, Charlotte Ferris, Christopher McGhee, Claire McNeill, Colin Bergin, Daniella Lynch, Fionnuala Lenehan, Gerry Gilleran, Gillian Lowe, Graham McIlroy, Helen Jenner, Helen Shackleford, Isma Younis, Jaspret Gill, Jimmy Musngi, Joanne Dasgin, Joanne Gresty, Joseph Nyaboko, Juneka Begum, Katerine Festejo, Katherine Lucas, Katie Price, Khushpreet Bhandal, Kristina Gallagher, Kyriaki Tsakiridou, Lauren Cooper, Louise Wood, Lulu Amutike, Marie Thomas, Marwan Kwok, Melanie Kelly, Michelle Bates, Nafeesah Ahmad Haider, Nicholas Adams, Oliver Topping, Rachel Smith, Rani Maria Joseph, Salma Kadiri, Samantha Caddick, Samuel Harrison, Shereef Elmoamly, Stavroula Chante, Sumaiyyah Gauhar, Syed Ashraf, Tabinda Kharodia, Zhane Peterkin, Isgro Graziella, Hakeem Yusuff, David Sutton, Ian Massey, Jade Di-Silvestro, Joanne Hiden, Mia Johnson, Richard Buka, Claire Lentaigne, Jackie Wooding, Nicola Crosbie, Ana Alvaro, Emma Drasar, Elen Roblin, Georgina Santiapillai, Kathryn Simpson, Kayleigh Gilbert, Yanrong Jiang, Zara Sayar, Zehraa Al-Khafaji

**Affiliations:** Centre for Haematology, Department of Immunology and Inflammation, Imperial College London; Department of Haematology, Imperial College Healthcare NHS Trust; Department of Computer Science, University of East London; National Heart and Lung Institute, Imperial College London, London; Institute of Cardiovascular Sciences, University of Birmingham, Edgbaston, Birmingham; Sheffield Teaching Hospitals NHS Foundation Trust, Department of Haematology, Royal Hallamshire Hospital, Broomhall, Sheffield, UK; Centre for Haematology, Department of Immunology and Inflammation, Imperial College London; Department of Haematology, Imperial College Healthcare NHS Trust

**Keywords:** autoimmune rheumatologic disease, COVID-19, mortality, thrombosis, bleeding, APS, SLE, RA

## Abstract

**Objective:**

To establish the demographic characteristics, laboratory findings and clinical outcomes in patients with autoimmune disease (AD) compared with a propensity-matched cohort of patients without AD admitted with COVID-19 to hospitals in the UK.

**Methods:**

This is a multicentre observational study across 26 NHS Trusts. Data were collected both retrospectively and prospectively using a predesigned standardized case record form. Adult patients (≥18 years) admitted between 1 April 2020 and 31 July 2020 were included.

**Results:**

Overall, 6288 patients were included to the study. Of these, 394 patients had AD prior to admission with COVID-19. Of 394 patients, 80 patients with SLE, RA or aPL syndrome were classified as severe rheumatologic AD. A higher proportion of those with AD had anaemia [240 (60.91%) *vs* 206 (52.28%), *P* = 0.015], elevated LDH [150 (38.08%) *vs* 43 (10.92%), *P* < 0.001] and raised creatinine [122 (30.96%) *vs* 86 (21.83%), *P* = 0.01], respectively. A significantly higher proportion of patients with severe rheumatologic AD had elevated CRP [77 (96.25%) *vs* 70 (87.5%), *P* = 0.044] and LDH [20 (25%) *vs* 6 (7.5%), *P* = 0.021]. Patients with severe rheumatologic AD had significantly higher mortality [32/80 (40%)] compared with propensity matched cohort of patients without AD [20/80 (25%), *P* = 0.043]. However, there was no difference in 180-day mortality between propensity-matched cohorts of patients with or without AD in general (*P* = 0.47).

**Conclusions:**

Patients with severe rheumatologic AD had significantly higher mortality. Anaemia, renal impairment and elevated LDH were more frequent in patients with any AD while elevated CRP and LDH were more frequent in patients with severe rheumatologic AD both of which have been shown to associate with increased mortality in patients with COVID-19.

Rheumatology key messages

- Demographic characteristics, laboratory findings and clinical outcomes in autoimmune disease patients developed COVID-19 were established.
- Patients with severe rheumatologic autoimmune (AD) disease had significantly higher mortality following COVID-19.
- Anaemia, renal impairment and elevated LDH were more frequent in patients with AD developed COVID-19.

## Introduction

Coronavirus disease 2019 (COVID-19) is a global pandemic leading to an unprecedented health crisis. The World Health Organization (WHO) declared the novel coronavirus outbreak to be a pandemic in March 2020. Although the number of patients with severe infection is gradually decreasing in some countries due to mass vaccination, it remains a global threat.

COVID-19 is associated with increased risk for thrombosis in addition to causing respiratory failure with or without multi-organ failure and death. Some studies found that patients with autoimmune and inflammatory conditions are at increased risk for COVID-19-associated hospitalizations and worse disease outcomes [1]. However, autoimmune diseases (ADs) are a broad category of diseases with differing severity, from requiring no treatment to multiple immunosuppressive treatments. It is likely that the clinical course and the outcomes of COVID-19 vary in patients with AD depending on the severity of the AD and the immunosuppressive treatment. There are >80 autoimmune conditions affecting >4 million people in the UK. ADs such as rheumatoid arthritis (RA), Systemic lupus erythematosus (SLE) and antiphospholipid syndrome (APS) are generally considered to be severe rheumatologic ADs associated with a higher risk of developing thrombosis in addition to their other complications [2]. In a propensity score–matched analysis from a nationwide multicentric research network study assessing the short-term outcome of COVID-19 patients with SLE, the mortality was comparable to that of the general population, but SLE patients had higher risks of hospitalization, admission to an intensive care unit (ICU), mechanical ventilation, stroke, venous thromboembolism (VTE) and sepsis [3]. Additionally, many studies have demonstrated a frequent occurrence of autoantibodies, including aPL, in patients with COVID-19 [4]. The prevalence of aPL was even higher in patients with severe disease, but there was no association between aPL positivity and disease outcomes including thrombosis, invasive ventilation and mortality. As transiently positive aPL is a well-known phenomenon in patients with acute infection, the significance of these antibodies remains to be determined [5], although some studies have demonstrated aPL from patients with COVID-19 caused thrombosis in a mouse model [6].

The aim of this study was to establish the demographic characteristics, laboratory findings and clinical outcomes in patients with AD compared with a propensity-matched cohort of patients with no AD admitted with COVID-19 to hospitals in the UK.

## Methods

This study is reported according to the Strengthening the Reporting of Observational Studies in Epidemiology statement.

### Study design, population and data collection

Coagulopathy associated with COVID-19 (CA-COVID-19) is a multicentre observational study across 26 NHS Trusts (listed in Supplementary Appendix pages 1–2, available at *Rheumatology* online) within the UK (https://clinicaltrials.gov/ct2/show/NCT04405232).

The study was approved by the Human Research Authority (HRA) and Health and Care Research Wales (HCRW) and the local Caldicott Guardian at Scotland (reference 20/HRA/1785).

We included adult patients (≥18 years) admitted to hospital during the first wave of the COVID-19 pandemic in the UK between 1 April 2020 and 31 July 2020. This article includes only patients with AD diagnosed prior to admission to a hospital with COVID-19 and an equal size propensity-matched cohort of patients with no AD with COVID-19 admitted to a hospital during the first wave of the COVID-19 pandemic (1 March–31 May 2020). All patients had severe acute respiratory syndrome coronavirus 2 confirmed by real-time PCR on nasopharyngeal swabs or lower respiratory tract aspirates.

### Data collection

Data were collected both retrospectively and prospectively using a predesigned standardized case record form (CRF) and entered in a central electronic database [Coagulopathy Associated with COVID-19 (CA-COVID-19)] (REDcap version 10.0.10; Vanderbilt University, Nashville, TN, USA) hosted by Imperial College London. At the time of writing the paper, all outcomes had been completed and no patient remained in hospital. As the data were collected by clinicians directly involved in patient care with no breach of privacy or anonymity by allocating a unique study number with no direct patient-identifiable data, consent was waived by the HRA. Baseline patient demographics, comorbidities, haematological and biochemical blood results on the day of admission and clinical outcomes until the day of discharge/death were collected. At the time of writing this paper, all patients had completed follow-up until day 180 post-hospital admission or death.

## Outcomes

The primary outcome was 180 day mortality. Secondary outcomes were thrombosis, major bleeding, the development of multiorgan failure (MOF) and ICU admission.

## Definitions of clinical outcomes

### Mortality

All-cause mortality was collected and classified as directly related to COVID-19, directly related to thrombosis, directly related to bleeding or related to other causes.

### Thrombosis and bleeding complications

Thrombosis and bleeding complications were identified on clinically indicated computerized tomography (CT) scan or ultrasound scan (US) imaging. Thrombotic events were defined as image-confirmed pulmonary embolism (PE), deep vein thrombosis (DVT) or arterial thrombosis. Bleeding events were defined as major or clinically relevant minor haemorrhages according to International Society on Thrombosis and Haemostasis classification [7] (Supplementary Table S1, available at *Rheumatology* online).

### Multi-organ failure

Multi-organ failure was defined as failure in two or more organ systems that required interventions to maintain homeostasis.

### Admission to an ICU

This was defined as patients who required continuous positive airway pressure ventilation (CPAP) or mechanical ventilation with or without extracorporeal membrane oxygenation or required other organ support.

## Statistical analysis

Propensity score matching was performed using the nearest neighbours method, with a desired ratio of 1:1 between patients with and without AD. Covariates (demographics and comorbidities) used for propensity score matching are summarized in Supplementary Fig. S1, available at *Rheumatology* online. Laboratory results at presentation were not included in the propensity matching. Factors for propensity matching were chosen based on factors found to contribute to increased mortality in published studies of patients with COVID-19. Propensity matching was performed for patients with any AD and for patients with severe rheumatologic AD separately. The characteristics of the treated and untreated patients were summarized and compared using descriptive statistics. The probability of survival between patients with and without AD were assessed using Kaplan–Meier curves. Characteristics of patients who had AD were compared with patients who did not have AD using the chi-squared or chi-squared trend test. Propensity score matching and analysis were performed using R (R Foundation for Statistical Computing, Vienna, Austria). Two-tailed *P*-values <0.05 were considered statistically significant.

## Results

**Fig. 1:**
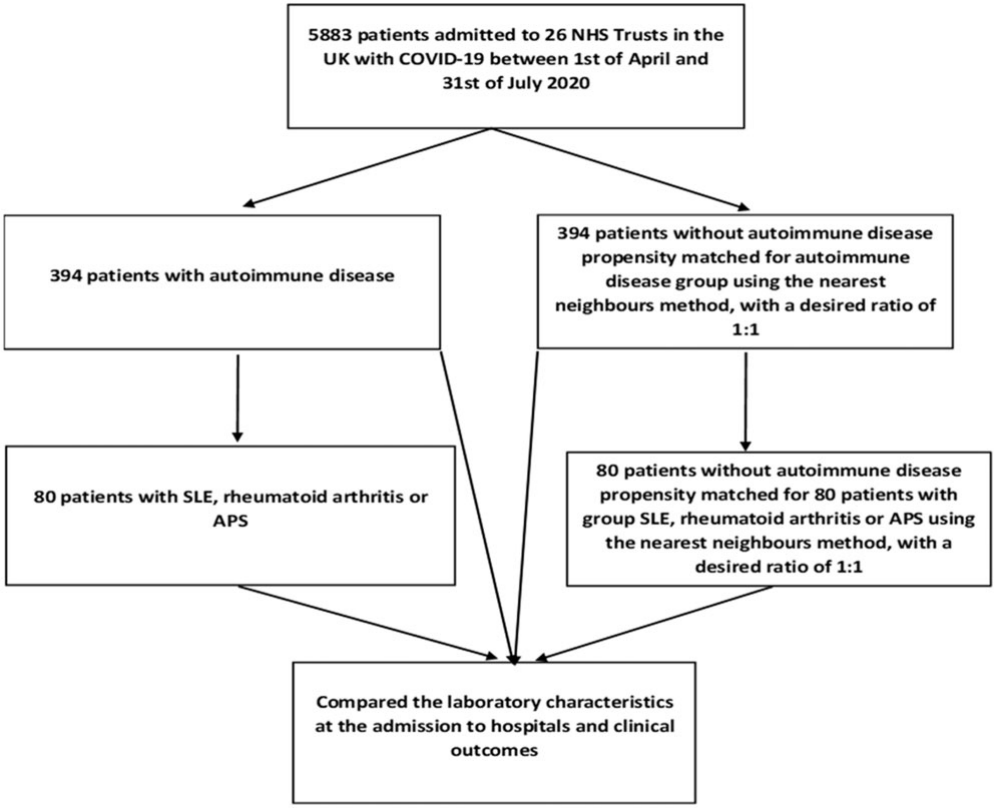
Inclusion of patients into the study and analysis plan

Overall, 6288 patients with COVID-19 were admitted to 26 NHS Trusts in the UK between 1 April and 31 July 2020. Of these patients, we analysed 394 classified as having AD prior to admission with COVID-19. The patients with AD group included those with chronic inflammatory arthritis, including RA, PsA and SpA; CTD, including SLE, SS, SSc and PMR; vasculitides and APS (Supplementary Table S2, available at *Rheumatology* online). Of 394 patients, 80 had SLE, RA or APS and were classified as having severe rheumatologic AD (Fig. 1). These patients are more likely to require immunosuppressive medication and be associated with an increased risk of thrombosis, which may cause severe complications when they develop COVID-19.

Of 80 patients classified as severe rheumatologic AD, 37 (46.2%) had RA, 34 (42.5%) had SLE and 9 (11.3%) had APS. Fifteen of 37 (40.5%) patients with RA were on methotrexate or other DMARDs, while 10/34 patients (29.4%) with SLE were on non-steroidal immunosuppressive drugs (mycophenolate mofetil and ciclosporin). Patients with APS were not on any immunosuppressive drugs, but 3/9 (33.5%) patients were on HCQ (Supplementary Table S3, available at *Rheumatology* online).

## All ADs compared with non-ADs prior to propensity matching

There was no age difference between patients with and without AD; the median age of patients with AD was 71 years (IQR 61–82) compared with 74 years (IQR 59–83) in patients without AD (*P* = 0.78). As expected, the majority of AD patients were female [229/394 (58.12%) *vs* 165/394 (41.88%); *P* < 0.001], although the majority of the patients admitted to hospitals with COVID-19 were male [3279/5894 (55.6%) male *vs* 2615/5894 (44.4%) female; *P* < 0.001]. There were no differences in BMI, ethnicity or comorbidities between patients with and without AD. The majority of patients with AD had below normal haemoglobin at the time of admission to hospital [240/394 (60.91%) *vs* 2895/5894 (49.12%); *P* < 0.001]. A higher proportion of patients with AD had elevated creatinine levels while a lower proportion had elevated prothrombin time (PT) compared with those without AD [creatinine above normal: 122/394 (30.96%) *vs* 1565/5894 (26.56%), *P* = 0.03; PT above normal: 4330/5894 (73.46%) *vs* 263/394 (66.75%), *P* = 0.004]. There were no differences in the other laboratory parameters, notably lactate dehydrogenase (LDH), CRP and D-dimer levels, between patients with and without AD at the time of admission to hospital with COVID-19. Patients’ characteristics, comorbidities and laboratory parameters at admission are summarized in Table 1.

**Table 1:** Clinical characteristics and admission laboratory parameters of patients with and without AD

Characteristics	Subgroup	No AD, *n* (%)	AD, *n* (%)	*P*-value^a^	Propensity-matched no AD, *n* (%)	*P*-value^b^
Overall		5894	394		394	
Patient gender	Male	3279 (55.6)	165 (41.88)	**<0.001**	165 (41.88)	1
	Female	2615 (44.4)	229 (58.12)		229 (58.12)	
Patient age (years)	≤29	143 (2.42)	10 (2.53)	0.87	6 (1.52)	0.94
	30–49	654 (11.10)	33 (8.38)		44 (11.17)	
	50–69	1639 (27.81)	122 (30.96)		120 (30.46)	
	70–89	2907 (49.32)	204 (51.78)		189 (47.97)	
	≥89	551 (9.35)	25 (6.35)		35 (8.88)	
BMI	≤18.5	124 (2.10)	17 (4.31)	0.87	11 (2.79)	0.96
	18.6–24.9	1629 (27.64)	93 (23.60)		99 (25.13)	
	25–29.9	2095 (35.54)	147 (37.31)		137 (34.77)	
	30–39.9	1806 (30.64)	121 (30.72)		120 (30.46)	
	≥40	240 (4.08)	16 (4.06)		27 (6.85)	
Ethnicity	White	4312 (73.16)	313 (79.44)	0.08	277 (70.30)	0.09
	Mixed multiple ethnic	32 (0.54)	4 (1.02)		3 (0.76)	
	Asian/Asian British	333 (5.65)	16 (4.06)		26 (6.60)	
	Black African/Caribbean	181 (3.07)	12 (3.05)		9 (2.28)	
	Other ethnic group	187 (3.17)	7 (1.78)		11 (2.79)	
	Unknown	849 (14.41)	42 (10.66)		68 (17.26)	
Previous history of VTE	No	5554 (94.23)	363 (92.13)	0.13	375 (95.18)	0.08
	Yes	340 (5.77)	31 (7.87)		19 (4.82)	
Malignancy	No	5272 (89.45)	353 (89.53)	0.99	359 (91.11)	0.55
	Yes	622 (10.55)	41 (10.47)		35 (8.89)	
Hypertension	No	3129 (53.08)	205 (52.03)	0.72	202 (51.27)	0.89
	Yes	2765 (46.92)	189 (47.97)		192 (48.73)	
Hypercholesterolemia	No	4978 (84.46)	324 (82.23)	0.27	320 (81.22)	0.78
	Yes	916 (15.54)	70 (17.77)		74 (18.78)	
Heart disease	No	4556 (77.30)	306 (77.66)	0.92	304 (77.16)	0.93
	Yes	1338 (22.70)	88 (22.34)		90 (22.84)	
Diabetes	No	4202 (71.29)	278 (70.56)	0.80	272 (69.03)	0.70
	Yes	1692 (28.71)	116 (29.44)		122 (30.97)	
History of smoking	None	2285 (39.11)	143 (36.39)	0.87	143 (36.39)	0.95
	Current smoker	280 (4.79)	22 (5.60)		22 (5.60)	
	Ex-smoker	1240 (21.22)	105 (26.71)		105 (26.71)	
	Unknown	2038 (34.88)	124 (31.30)		124 (31.30)	
Liver disease	No	5687 (96.49)	370 (93.91)	0.09	376 (95.43)	0.43
	Yes	207 (3.51)	24 (6.09)		18 (4.57)	
Lung disease	No	4457 (75.62)	286 (72.59)	0.2	286 (72.59)	1
	Yes	1437 (24.38)	108 (27.41)		108 (27.41)	
Existing renal failure	No	4839 (82.10)	314 (79.70)	0.26	318 (80.71)	0.79
	Yes	1055 (17.90)	80 (20.30)		76 (19.29)	
Antiplatelet therapy prior to admission	No	4794 (81.34)	314 (79.70)	0.46	320 (81.22)	0.65
	Yes	1100 (18.66)	80 (20.30)		74 (18.78)	
Ferritin, μg/L	Below normal (<20)	19 (0.30)	0 (0)	0.40	1 (0.25)	0.87
	Normal (20–186)	191 (3.24)	19 (4.8)		16 (4.06)	
	Above normal (>186)	5684 (96.36)	375 (95.20)		377 (95.69)	
Lactate, mmol/L	Normal (<2.1)	5220 (88.56)	353 (89.59)	0.519	354 (89.85)	0.907
	Above normal (>2.1)	674 (11.44)	41 (10.41)		40 (10.15)	
Haemoglobin [men (women)], g/L	Below normal <130 (<115)	2895 (49.12)	240 (60.91)	<0.001	206 (52.28)	0.015
	Normal 130-160 (115–150)	2670 (45.3)	138 (35.02)		166 (42.13)	
	Above normal >160 (>150)	329 (5.58)	16 (4.07)		22 (5.59)	
Troponin, ng/L	Normal (<19.8)	1764 (29.93)	126 (31.98)	0.399	120 (30.46)	0.645
	Above normal (>19.7)	4130 (70.07)	268 (68.02)		274 (69.54)	
LDH, IU/L	Below normal (<266)	165 (2.80)	12 (3.04)	0.99	19 (4.82)	<0.001
	Normal (266-500)	3446 (58.47)	232 (58.88)		332 (84.26)	
	Above normal (>500)	2283 (38.73)	150 (38.08)		43 (10.92)	
Prothrombin time, sec	Below normal (<10.2)	76 (1.29)	9 (2.28)	0.004	6 (1.52)	0.092
	Normal (10.2-13.2)	1488 (25.25)	122 (30.96)		104 (26.40)	
	Above normal (>13.2)	4330 (73.46)	263 (66.75)		284 (72.08)	
APTT, sec	Below normal (<26.0)	585 (9.92)	50 (12.69)	0.15	30 (7.61)	0.23
	Normal (26–36)	4568 (77.50)	299 (75.88)		318 (80.71)	
	Above normal (>36.0)	741 (12.58)	45 (11.42)		46 (11.68)	
Platelets, ×10^9^/L	Below normal (<150)	1001 (16.98)	61 (15.48)	0.319	71 (18.02)	0.567
	Normal (150–400)	4459 (75.65)	300 (76.14)		288 (73.10)	
	Above normal (>400)	434 (7.36)	33 (8.38)		35 (8.89)	
WBCs, ×10^9^/L	Below normal (<4.1)	542 (9.20)	36 (9.14)	0.92	43 (10.91)	0.368
	Normal (4.1–11.1)	4019 (68.19)	268 (68.02)		268 (68.02)	
	Above normal (>11.1)	1333 (22.61)	90 (22.84)		83 (21.07)	
Neutrophils, ×10^9^/L	Below normal (<2.1)	249 (4.22)	17 (4.31)	0.654	16 (4.06)	0.185
	Normal (2.1–6.7)	3126 (53.04)	203 (51.52)		226 (57.36)	
	Above normal (>6.7)	2519 (42.74)	174 (44.16)		152 (38.58)	
Lymphocytes, µL	Below normal (<1.3)	4484 (76.08)	299 (75.89)	0.938	286 (72.59)	0.29
	Normal (1.3–3.7)	1409 (23.91)	95 (24.11)		108 (27.41)	
	Above normal (>3.7)	1 (0.01)	0 (0)		0 (0)	
Fibrinogen, g/L	Below normal (<1.5)	128 (2.17)	10 (25.38)	0.929	8 (2.03)	0.353
	Normal (1.5–4.5)	593 (10.06)	36 (9.14)		51 (12.94)	
	Above normal (>4.5)	5173 (87.77)	348 (88.32)		335 (85.02)	
ALT, IU/L	Below normal (<8)	120 (2.04)	13 (3.30)	0.1	10 (2.54)	0.2
	Normal (8–40)	3988 (67.66)	267 (67.76)		264 (67.0)	
	Above normal (>40)	1786 (30.30)	114 (28.93)		120 (30.46)	
Bilirubin, µmol/L	Normal (0–20)	5293 (89.80)	356 (90.36)	0.720	353 (89.59)	0.724
	Above normal (>20)	601 (10.20)	38 (9.64)		41 (10.41)	
Creatinine, µmol/L	Below normal (<60)	833 (14.13)	67 (17.01)	0.03	56 (14.21)	0.01
	Normal (60–120)	3496 (59.31)	205 (52.03)		252 (63.96)	
	Above normal (>120)	1565 (26.56)	122 (30.96)		86 (21.83)	
CRP, mg/L	Normal (0–10)	571 (9.68)	30 (7.61)	0.137	44 (11.17)	0.088
	Above normal (>10)	5323 (90.31)	364 (92.39)		350 (88.83)	
D-dimer, ng/ml	Normal (0–500)	445 (7.55)	35 (8.88)	0.367	33 (8.38)	0.8
	Above normal (>500)	5449 (92.45)	359 (9.11)		361 (91.62)	

a *P*-value refers to the comparison of the AD *vs* no AD groups.

b *P*-value refers to the comparison of the AD group and the propensity-matched AD group. *P*-values <0.05 are shown in bold.

APTT: activated partial thromboplastin time; WBCs: white blood cells; ALT: alanine transferase.

### All AD patients compared with non-AD patients after propensity matching

As expected, there were no differences in the demographics and comorbidities of the patients with and without AD after propensity matching (Table 1). However, even after propensity matching, a higher proportion of patients with any AD had low haemoglobin compared with patients without AD [240 (60.91%) *vs* 206 (52.28%); *P* = 0.015]. Furthermore, a higher proportion of patients with AD had elevated LDH and creatine levels [LDH in 150 (38.08%) *vs* 43 (10.92%), *P* < 0.001; creatinine in 122 (30.96%) *vs* 86 (21.83%), *P* = 0.01]. There were no differences in the other laboratory parameters between the two groups (Table 1).

## Patients with severe rheumatologic AD

Comparisons were made between the 80 patients classified as severe rheumatologic AD with a 1:1 propensity matched cohort of patients without AD. As expected, no differences were seen in patient demographics and comorbidities between the two groups following the propensity matching. In patients with severe rheumatologic AD, the female preponderance was higher than in the all-AD group [55/80 (68.75%) female *vs* 25/80 (31.25%) male] (Table 2). Furthermore, a significantly higher proportion of patients with severe rheumatologic AD had elevated CRP and LDH levels compared with patients without AD [CRP in 77 (96.25%) *vs* 70 (87.5%), *P* = 0.044; LDH in 20 (25%) *vs* 6 (7.5%), *P* = 0.021]. There were no differences in the other laboratory parameters between the two groups (Table 2).

**Table 2:** Clinical characteristics and admission laboratory parameters of patients with or without severe rheumatologic AD

Characteristics	Subgroup	Severe AD, *n* (%)	Propensity-matched no AD, *n* (%)	*P*-value
Overall		80	80	
Patient gender	Male	25 (31.25)	25 (31.25)	1
	Female	55 (68.75)	55 (68.75)	
Patient age (years)	≤29	2 (2.5)	2 (2.5)	0.587
	30–49	0 (0)	0 (0)	
	50–69	25 (31.25)	20 (25)	
	70–89	48 (60)	46 (57.5)	
	≥89	5( 6.25)	12 (15)	
BMI	≤18.5	4 (5)	2 (2.5)	0.587
	18.6–24.9	17 (21.25)	29 (36.25)	
	25–29.9	27 (33.75)	29 (36.25)	
	30–39.9	30 (37.5)	17 (21.25)	
	≥40	2 (2.5)	3 (3.75)	
Ethnicity	White	66 (82.5)	60 (75)	0.269
	Mixed multiple ethnic	0 (0)	1 (1.25)	
	Asian/Asian British	2 (2.5)	3 (3.75)	
	Black African/Caribbean	2 (2.5)	0 (0)	
	Other ethnic group	0 (0)	1 (1.25)	
	Unknown	6 (12.5)	15 (18.75)	
Previous history of VTE	No	79 (98.75)	77 (96.25)	0.734
	Yes	1 (1.25)	3 (3.75)	
Malignancy	No	68 (85)	71 (88.75)	0.486
	Yes	12 (15)	9 (11.25)	
Hypertension	No	45 (56.25)	46 (57.5)	0.874
	Yes	35 (43.75)	34 (42.5)	
Hypercholesterolemia	No	69 (86.25)	71 (88.75)	0.079
	Yes	11 (13.75)	9(11.25)	
Heart disease	No	62 (77.5)	64 (80)	0.701
	Yes	18 (22.5)	16 (20)	
Diabetes	No	59 (73.75)	61 (76.25)	0.717
	Yes	21 (26.25)	19 (23.75)	
History of smoking	None	32 (40)	28 (35.45)	0.230
	Current smoker	3 (3.75)	4 (5.06)	
	Ex-smoker	26 (32.5)	15 (19.99)	
	Unknown	19 (23.75)	32 (40.5)	
Liver disease	No	79 (98.75)	78 (97.5)	0.563
	Yes	1 (1.25)	2 (2.5)	
Lung disease	No	53 (66.25)	54 (67.5)	0.868
	Yes	27 (33.75)	26 (32.5)	
Existing renal failure	No	68 (85)	65 (81.25)	0.530
	Yes	12 (15)	15 (18.75)	
Antiplatelet therapy prior to admission	No	61 (76.25)	62 (77.5)	0.852
	Yes	19 (23.75)	18 (22.5)	
Ferritin, ug/L	Below normal (<20)	0 (0)	2 (2.5)	0.587
	Normal (20–186)	4 (5)	2 (2.5)	
	Above normal (>186)	76 (95)	86 (95)	
Lactate, mmol/L	Normal (<2.1)	70 (87.5)	73 (91.25)	0.445
	Above normal (>2.1)	10 (12.5)	7 (8.75)	
Haemoglobin [men (women)], g/L	Below normal <130 (<115)	24 (30)	17 (21.25)	0.269
	Normal 130–160 (115–150)	49 (61.25)	55 (68.75)	
	Above normal >160 (>150)	7 (8.75)	8 (10)	
Troponin, ng/L	Normal (<19.8)	20 (25)	23 (27.75)	0.595
	Above normal (>19.7)	60 (75)	57 (71.25)	
LDH, IU/L	Below normal (<266)	3 (3.75)	1 (1.25)	**0.021**
	Normal (266–500)	57 (71.25)	73 (91.25)	
	Above normal (>500)	20 (25)	6 (7.5)	
Prothrombin time, sec	Below normal (<10.2)	0 (0)	1 (1.25)	0.143
	Normal (10.2–13.2)	21 (26.25)	19 (23.75)	
	Above normal (>13.2)	59 (73.75)	60 (75)	
APTT, sec	Below normal (<26.0)	8 (10)	8 (10)	0.508
	Normal (26–36)	60 (75)	64 (80)	
	Above normal (>36.0)	12 (15)	8 (10)	
Platelets, ×10^9^/L	Below normal (<150)	14 (17.5)	13 (16.25)	0.875
	Normal (150–400)	59 (73.75)	60 (75)	
	Above normal (>400)	7 (8.75)	7 (8.75)	
WBCs, ×10^9^/L	Below normal (<4.1)	6 (7.5)	5 (6.25)	0.761
	Normal (4.1–11.1)	57 (71.25)	57 (71.25)	
	Above normal (>11.1)	17 (21.25)	18 (22.5)	
Neutrophils, ×10^9^/L	Below normal (<2.1)	3 (3.75)	2 (2.5)	0.667
	Normal (2.1–6.7)	42 (5.25)	47 (58.75)	
	Above normal (>6.7)	35 (43.75)	31 (38.75)	
Lymphocytes, µL	Below normal (<1.3)	62 (77.5)	59 (73.75)	0.584
	Normal (1.3–3.7)	18 (22.5)	21 (26.25)	
	Above normal (>3.7)	0 (0)	0 (0)	
Fibrinogen, g/L	Below normal (<1.5)	2 (2.5)	1 (1.25)	0.327
	Normal (1.5–4.5)	5 (6.25)	12 (15)	
	Above normal (>4.5)	73 (91.25)	67 (83.75)	
ALT, IU/L	Below normal (<8)	2 (2.5)	2 (2.5)	0.863
	Normal (8-40)	61 (76.25)	60 (75)	
	Above normal (>40)	17 (21.25)	18 (2.25)	
Bilirubin, µmol/L	Normal (0–20)	75 (93.75)	73 (91.25)	0.551
	Above normal (>20)	5 (6.25)	7 (8.75)	
Creatinine, µmol/L	Below normal (<60)	22 (27.5)	16 (20)	0.308
	Normal (60–120)	47 (58.75)	51 (63.75)	
	Above normal (>120)	11 (13.75)	13 (16.25)	
CRP, mg/L	Normal (0–10)	3 (3.75)	10 (12.5)	**0.044**
	Above normal (>10)	77 (96.25)	70 (87.5)	
D-dimer, ng/ml	Normal (0–500)	5 (6.25)	6 (7.5)	0.757
	Above normal (>500)	75 (93.75)	74 (92.5)	

*P*-values <0.05 are shown in bold.

APTT: activated partial thromboplastin time; WBCs: white blood cells; ALT: alanine transferase.

## Outcomes in patients with any AD compared with non-AD after propensity matching

**Fig. 2:**
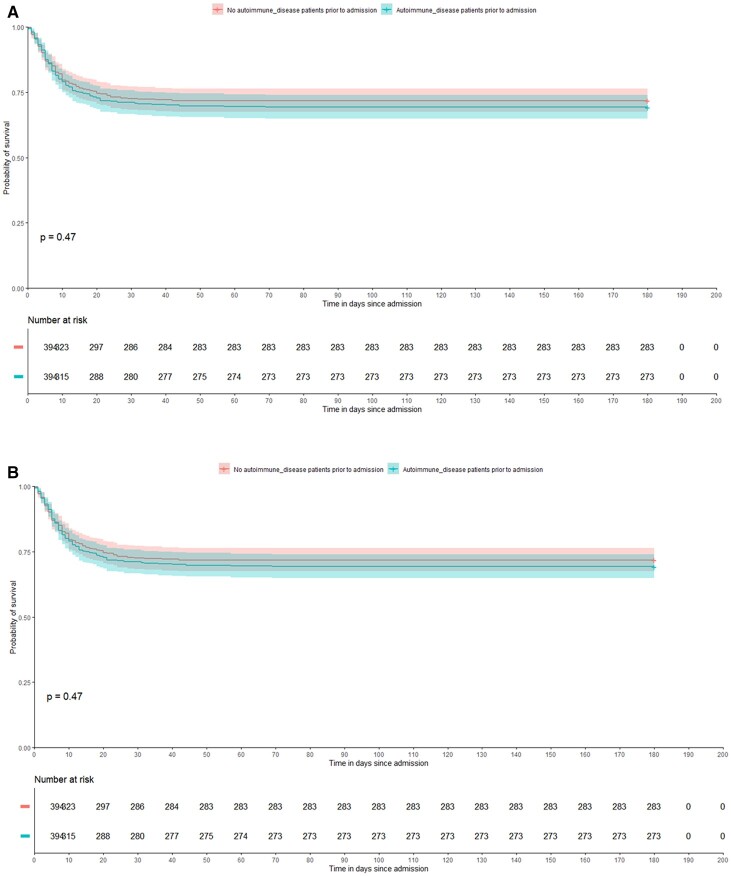
Probability of 180 day survival in patients with and without AD (**A**) Probability of 180 day survival in patients with AD *vs* no AD admitted with COVID-19. (**B**) Probability of 180 day survival in patients classified as severe AD *vs* no AD admitted with COVID-19.

For the primary outcome, there was no difference in the 180 day mortality between the propensity-matched cohort of all patients with and without AD. The overall mortality in patients with any AD was 121/304 (30.71%) compared with 111/394 (28.17%) in patients with no AD (*P* = 0.435) (Fig. 2A).

For the secondary outcomes, there were no differences observed in the rate of thrombosis, major bleeding, the development of MOF or admission to an ICU in patients with any AD compared with those with no AD. There was a trend towards more patients with AD supported with CPAP [29/393 (7.36%) *vs* 17/394 (4.31%); *P* = 0.068] (Table 3).

**Table 3:** Medical interventions and clinical outcomes in patients with or without AD

Interventions	AD, *n* (%)	Propensity-matched patients with no AD, *n* (%)	*P*-value
CPAP	29 (7.36)	17 (4.31)	0.068
Mechanical ventilation	27 (6.85)	38 (9.64)	0.155
Antiplatelet treatment	27 (6.85)	25 (6.35)	0.774
Thromboprophylaxis on admission	206 (52.28)	201 (51.01)	0.722
Thromboprophylaxis on discharge	25 (6.35)	22 (5.58)	0.652
Thrombolysis	2 (0.5)	0 (0)	0.158
IVIG	1 (0.2)	2 (0.5)	0.563
Tocilizumab	1 (0.2)	1 (0.2)	1
Steroids	82 (20.81)	40 (10.15)	<0.001
Haemostatic support	6 (1.52)	7 (1.78)	0.780
Outcomes			
Renal failure	10 (2.54)	13 (3.30)	0.526
HIT	1 (0.2)	1 (0.2)	1
Minor bleeding	10 (2.54)	3 (0.76)	0.050
Major bleeding	12 (3.04)	9 (2.29)	0.508
Venous thrombosis	17 (4.31)	15 (3.80)	0.718
Arterial thrombosis	7 (1.78)	6 (1.52)	0.780
Multi-organ failure	10 (2.54)	11 (2.79)	0.825
Secondary infection	65 (16.49)	64 (16.24)	0.923
Death	121 (30.71)	111 (28.17)	0.435
Hospital-associated thrombosis	2 (0.5)	1 (0.2)	0.564

HIT: heparin-induced thrombocytopenia.

### Outcomes in patients with severe rheumatologic AD compared with non-AD after propensity matching

For the primary outcome, in contrast to patients with any AD, those with severe rheumatologic AD had significantly higher mortality [all-cause mortality; 32/80 (40%)] compared with the propensity-matched cohort of patients with no AD [20/80 (25%), *P* = 0.043] (Fig. 2B). There was a trend towards higher mortality in patients with severe rheumatologic AD [40% (32/80)] compared with patients with other AD [28.3% (89/314); *P* = 0.056]. Secondary outcomes were similar; no differences were observed in the rate of thrombosis, major bleeding, the development of MOF or admission to an ICU in patients with severe rheumatologic AD compared with those with no AD (Table 4).

**Table 4:** Medical interventions and clinical outcomes in patients with and without severe rheumatologic AD

Interventions	Severe rheumatologic AD, *n* (%)	No AD, *n* (%)	*P*-value
CPAP	8 (10)	6 (7.5)	0.579
Mechanical Ventilation	6 (7.5)	4 (5)	0.517
Antiplatelet agent	5 (6.25)	7 (8.75)	0.551
Thromboprophylaxis on admission	46 (57.5)	42 (52.5)	0.528
Thromboprophylaxis on discharge	5 (6.25)	3 (3.75)	0.471
Thrombolysis	0 (0)	1 (1.25)	0.320
IVIG	0 (0)	1(1.25)	0.320
Tocilizumab	1 (1.25)	0 (0)	0.320
Steroids	18 (22.5)	5 (6.25)	0.003
Haemostatic support	2 (2.5)	1 (1.25)	0.563
Outcomes			
Renal failure	3 (3.75)	3 (3.75)	1
HIT	0 (0)	0 (0)	NA
Minor bleeding	1 (1.25)	1 (1.25)	1
Major bleeding	3 (3.75)	1 (1.25)	0.315
Venous thrombosis	2 (2.5)	5 (6.25)	0.249
Arterial thrombosis	0 (0)	0 (0)	NA
Multi-organ failure	4 (5)	1 (1.25)	0.176
Secondary Infection	16 (20)	9 (11.25)	0.129
Death	32 (40)	20 (25)	0.043
Hospital-associated thrombosis	1 (1.25)	1 (1.25)	1

HIT: heparin-induced thrombocytopenia.

## Clinical interventions

There were no differences in the clinical interventions during the hospital admission in patients with or without AD as a whole group or with severe rheumatologic AD, except a significantly higher proportion of patients with any AD or severe rheumatologic AD received steroids compared with patients with no AD [82/394 (20.81%) *vs* 40/394 (10.15%), *P* < 0.001 and 18/80 (22.5%) *vs* 5/80 (6.25%), *P* = 0.003, respectively] (Table 3 for any AD and Table 4 for severe rheumatologic AD).

## Discussion

In this large multicentre observational study across the UK assessing the clinical characteristics and outcomes of patients with any AD and those with severe rheumatologic AD, we found that the presence of any AD did not increase the risk of mortality or other outcomes (thrombosis, major bleeding, MOF or admission to an ICU) compared with the propensity-matched cohort of patients with no AD. However, patients classified as severe rheumatologic AD (SLE, RA or APS) had significantly higher mortality compared with patients with no AD. No differences were seen in the secondary outcomes between the two groups. Following propensity matching for demographics and comorbidities, a higher proportion of patients with AD had low haemoglobin and elevated LDH and creatine levels compared with patients with no AD. In those with severe rheumatologic AD, elevated CRP and LDH were more common compared with patients without AD. Generally, ADs are more common in women, occurring at a ratio of 2:1 [8], whereas the COVID-19 disease severity and admission rate are higher in men [9]. These differences were preserved in this study.

ADs are a heterogeneous group of conditions typified by dysregulation of the immune system. Most of the patients with AD received or were receiving immunosuppressive medications, which make them more susceptible to infections and complications. Observational studies assessing the risk of acquiring COVID-19 and outcomes in patients with AD reported conflicting results. A cross-sectional study in northeast Italy reported that patients with AD had a similar rate of COVID-19 compared with the general population [10]. Another Italian study also found that the presence of AD did not increase the risk of COVID-19 [11]. Furthermore, they suggested that the outcome of patients with AD did not differ from patients with no AD [11]. However, this study did not perform propensity matching for the study groups, which, as shown in this study, are significantly different in important respects. In contrast, the results of a multicentre retrospective study from China showed that patients with AD might be more susceptible to COVID-19 compared those without [12]. Additionally, a Spanish study that assessed the association between the outcome and the potential prognostic variables, adjusted by COVID-19 treatment in patients with AD compared with a matched cohort (for sex and age and blinded to outcome or other variables but not propensity matching for all comorbidities) of patients with no AD, reported that hospitalized patients with AD have a more severe course [13]. In the current propensity-matched study, we did not observe a difference in the mortality or secondary outcomes between patients with any AD compared with patients with no AD (Table 3). This could be due a higher proportion of patients with any AD being given steroids, which has been shown to improve mortality in patients with COVID-19 [14]. However, the mortality rate was still significantly higher in patients with severe rheumatologic AD despite a higher proportion receiving steroids. Additionally, there was a trend towards higher mortality in patients classified as severe rheumatologic AD compared with patients with other ADs (*P* = 0.056). The higher mortality in patients with severe rheumatologic AD could indicate that these patients suffer more severe rheumatologic COVID-19, although no differences were seen in the secondary outcomes, including the rate of thrombosis, major bleeding, development of MOF or admission to an ICU. Therefore the cause for increased mortality in patients with severe rheumatologic AD was not clear. It is possible that prior non-steroidal immunosuppressive drugs contributed to the increased mortality in these patients (Supplementary Table S3, available at *Rheumatology* online).

Anaemia is a frequent complication in patients with AD. It is generally classified as anaemia of chronic disease and is usually multifactorial. Despite propensity matching for demographics and comorbidities, a higher proportion of patients with any autoimmune disease had anaemia on admission to hospital. However, a significantly higher proportion of patients with AD had elevated LDH, which could be due to ongoing tissue damage associated with AD and in some cases autoimmune haemolytic anaemia. Elevated CRP, a marker of disease severity in many ADs, was observed in a significantly higher proportion of patients with severe rheumatologic AD upon hospital admission compared with patients without AD. Both elevated CRP and LDH on admission are considered predictors of increased mortality in patients with COVID-19 [15, 16] and indeed these patients had a higher mortality rate compared with the control group in this study. Finally, serum creatinine was elevated on admission in a higher proportion of patients with AD. Renal failure is a frequent complication in these individuals and may additionally contribute to anaemia.

The main limitation of this study is that some of the data were collected retrospectively, but relevant information and clinical outcomes were recorded directly using a predefined, well-structured electronic CRF. However, this did not include DASs such as SLEDAI or the 28-joint DAS. The classification of RA, SLE and APS as severe rheumatologic AD compared with other ADs in the study may be regarded as arbitrary. It is possible that disease severity of any given AD at the time of admission with COVID-19 has an impact on primary or secondary outcomes beyond the primary AD diagnosis and immunosuppressive medications. As the disease severity scores were not included in the data collection, we were unable to assess the impact of the individual disease severity in the clinical outcomes in this study. Although the number of patients included in the study is relatively small, it comprises patients admitted to 26 NHS Trusts across the UK, providing a representative view of AD in the UK.

In conclusion, we found no differences in the clinical outcomes in patients with any AD compared with patients with no AD admitted to hospitals with COVID-19 from the first wave of the pandemic. However, those with severe rheumatologic AD had significantly higher mortality. Anaemia, renal impairment and elevated LDH were more frequent in patients with any AD, while elevated CRP and LDH were more frequent in patients with severe rheumatologic AD. Although vaccination has reduced the risk of mortality associated with COVID-19, patients with severe rheumatologic AD need additional attention if admitted to hospital with COVID-19.

## Supplementary Material

keac209_Supplementary_Data

## Data Availability

The data underlying this article will be shared upon reasonable request to the corresponding author. CA-COVID19 Study Collaborators: Aneurin Bevan University Health Board: Amanda Dell, Angela Hall, Anna Roynon, Anne Heron, Cheri Price, Claire Price, Clare Westacott, Debra Barnett, Gail Marshall, Gemma Hodkinson, Georgia Mallison, Grace Okoro, Joshua Gwatkin, Kirstin Davies, Lucy Shipp, Maxine Nash, Rhian Hughes, Rina Mardania and Sarah Lewis Sean Cutler; Aberdeen Royal Infirmary: Caroline Allan; Barts Health NHS Trust: Atiqa Miah, Dide Okaygun, Dan Hart, Faith Dzumbunu, James Leveson, Karen Torre, Louise Taylor, Priyanka Raheja, Sara Mamsa and Tasnima Ferdousi; Buckinghamshire Healthcare NHS Trust: Angharad Everden, Alice Ngumo, Doaa Ahmed, Efstathia Venizelou, James Herdman, Janice Carpenter, Konrad Bartkiewicz, Rebecca Cash and Renu Riat; Cardiff and Vale University Health Board: Abigail Downing, Ana Guerrero, Astrid Etherington, Chapa Gamage, Dilupa Gunasekara, Lee Morris, Raza Alikhan, Rebecca Cloudsdale, Samya Obaji, Stuart Cunningham and Sylvain Ndjombo; County Durham and Darlington NHS Foundation Trust: Amanda Cowton, Ami Wilkinson, Andrea Kay, Anne Sebakungu, Anne Thomson, Clare Brady, Dawn Egginton, Ellen Brown, Enid Wright, Gill Rogers, Hannah Plaschkes, Jacqui Jennings, Julie O’Brien, Julie Temple, Kathryn Potts, Kimberly Stamp, Kelly Postlethwaite, Louise Duncan, Margaret Randall, Mark Birt, Melanie Kent, Philip Mounter, Shelly Wood, Nicola Hewitson, Noreen Kingston, Susan Wadd, Sarah McAuliffe, Stefanie Hobson, Susan Riley, Suzanne Naylor and Vicki Atkinson; Cwm Taf Morgannwg University Health Board: Alysha Hancock, Bethan Deacon, Carla Pothecary, Caroline Hamilton, Ceri Lynch, Cerys Evenden, Deborah Jones, Ellie Davies, Felicity Page, Gareth Kennard-Holden, Gavin John, Joanne Pugh, Joelle Pike, Justyna Mikusek, Kevin Agravante, Kia Hancock, Lauren Geen, Meryl Rees and Natalie Stroud; Gateshead Health NHS Foundation Trust: Amanda Grahamslaw, Amanda Sanderson, Beverley McClelland, Caitlin Barry, Elaine Siddle, Lorraine Pearce, Lucy Blackwell, Maria Bokhari, Maureen Armstrong, Wendy Stoker and Wendy McCormick; Guy’s and St Thomas’ NHS Foundation Trust: Caterina Vlachou, Ben Garfield, Mihaela Gaspar, Maurizio Passariello, Paolo Bianchi and Stephane Ledot; Hampshire Hospitals NHS Foundation Trust: Aileen Madlin, Kerrianne Everard, Khushboo Panwar, Natasha Beacher, Niamh Cole, Sarah Mangles, Tamara Everington and Udaya Reddy; Imperial College Healthcare NHS Trust: Alka Shah, Anna Weatherill, Anthi Maropoulou, Bhagya Herath, Billy Hopkins, Camelia Vladescu, Caroline Ward, Christina Crossette-Thambiah Donna Copeland, Emily Pickford, Gaurika Kapoor, Isabella Lo, John Kilner, Keith Boland, Melanie Almonte, Neil Simpson, Niamh Bohnacker, Omolade Awomolo, Roochi Trikha, Samina Hussain, Serah Duro, Sophie Kathirgamanathan, Yasmine Needham, Yee Hui and Zainab Alashe; King’s College Hospital NHS Foundation Trust: Adrienne Abioye, Aileen Miranda, Christina Obiorah, Cynthia Dzienyo, Hasina Mangal, Hernan Zorraquino, Lara N. Roberts, Mariusz Racz, Maclaine Hipolito Johnson, Rachel Ryan, Tamara Swales, Tatiana Taran and Zoe Renshaw; Newcastle Hospitals NHS Foundation Trust: Alexander Langridge, Benjamin Evans, Callum Weller, Claire Judd, Douglas Jerry, Euan Haynes, Fatima Jamil, Ian McVittie, John Hanley, Julie Parker, Kayleigh Smith, Keir Pickard, Laura Kennedy, Meghan Acres, Mikaela Wiltshire, Nitha Ramachandran, Paul McAlinden, Paula Glancy, Smeera Nair, Tarek Almugassabi and Thomas Jarvis; NHS Grampian: Amanda Coutts, Andrew Laurie, Deborah Owen, Ian Scott, Jamie Cooper, Leia Kane, Lucy Sim, Mahmoud Abdelrahman and Victoria Poulton; Norfolk and Norwich University Hospitals NHS Foundation Trust: Jessica Griffin, Ria Markwell and Suzanne Docherty; North Cumbria Integrated Care NHS Foundation Trust: Alexander Brown, Barbara Cooper, Beverley Wilkinson, Diane Armstrong, Grace Fryer, Jane Gregory, Katherine Davidson, Melanie Clapham, Nicci Kelsall, Patricia Nicholls, Rachel Hardy, Roderick Oakes, Rosemary Harper, Sara Abdelhamid, Theresa Cooper, Una Poultney and Zoe Saunders; North Tees and Hartlepool NHS Foundation Trust: Alex Ramshaw, Alison Chilvers, Barbara Jean Campbell, Carol Adams, Claire Riley, Deborah Wilson, Helen Wardle, Jill Deane, Jill Skelton, Julie Quigley, Leigh Pollard, Liz Baker, Lynda Poole, Maria Weetman, Michele Clark, Nini Aung, Rachel Taylor, Sarah Rowling, Sarah Purvis and Vicky Collins: Northumbria Healthcare NHS Foundation Trust: Amy Shenfine, Catherine Ashbrook-Raby, Charlotte Bomken, Claire Walker, Faye Cartner, Helen Campbell, Jane Luke, Jessica Reynolds, Mari Kilner, Laura Winder, Linda Patterson, Lisa Gallagher, Nicola McLarty, Sandra Robinson, Steve Dodds, Toni Hall and Victoria Wright; Oxford University Hospitals NHS Foundation Trust: Agnes Eordogh, Alexandros Rampotas, Anna Maria Sanigorska, Christopher Deane, Kristine Santos, Olivia Lecocq, Rochelle Lay and Simon Fletcher; Royal Free London NHS Foundation Trust: Anna Tarnakina, Aniqa Tasnim, Anja Drebes, Cecilia Garcia, Elsa Aradom, Mariarita Peralta, Michaella Tomlin, Pratima Chowdary, Ramona Georgescu, Suluma Mohamed and Upuli Dissanayake; Royal Liverpool and Broadgreen University Hospitals NHS Trust: Carol Powell, James Doolan, Jessica Kenworthy, Joanne Bell, Lewis Jones, Mikiko Wilkinson, Rebecca Shaw, Ryan Robinson, Saman Mukhtar, Shane D’Souza, Tina Dutt and Tracy Stocks; Royal Papworth Hospital NHS Foundation Trust: Joshua Wade, Lenka Cagova, Maksym Kovzel and Rachel Jooste; Sheffield Teaching Hospitals NHS Foundation Trust: Alison Delaney and Claire Mapplebeck; South Tees NHS Foundation Trust: Alycon Walker, Andrea Watson, Andrew Vaux, Asia Sawar, Carol Hannaway, Charlotte Jacobs, Claire Elliot, Claire Elliott, Craig Mower, Daiana Ferro, Emanuela Mahmoud, Gill Laidlaw, Julie Potts, Keith Harland, Laura Munglani, Lauren Fall, Leanne Murray, Lesley Harris, Lisa Wayman, Lisa Westwood, Louisa Watson, Lynne Naylor, Matthew Siddaway, Paula Robson, Rita Mohan, Sarah Essex, Sara Griffiths and Steven Liggett; University Hospital Southampton NHS Foundation Trust: Andreia Valente, Rashid Kazmi, Ruth Kirby, Sarah Bowmer and Yanli Li; University Hospitals Birmingham NHS Foundation Trust: Alice Longe, Amy Bamford, Anand Lokare, Andrew McDarby, Aneta Drozd, Cathy Stretton, Catia Mulvihill, Charlotte Ferris, Christopher McGhee, Claire McNeill, Colin Bergin, Daniella Lynch, Fionnuala Lenehan, Gerry Gilleran, Gillian Lowe, Graham McIlroy, Helen Jenner, Helen Shackleford, Isma Younis, Jaspret Gill, Jimmy Musngi, Joanne Dasgin, Joanne Gresty, Joseph Nyaboko, Juneka Begum, Katerine Festejo, Katherine Lucas, Katie Price, Khushpreet Bhandal, Kristina Gallagher, Kyriaki Tsakiridou, Lauren Cooper, Louise Wood, Lulu Amutike, Marie Thomas, Marwan Kwok, Melanie Kelly, Michelle Bates, Nafeesah Ahmad Haider, Nicholas Adams, Oliver Topping, Rachel Smith, Rani Maria Joseph, Salma Kadiri, Samantha Caddick, Samuel Harrison, Shereef Elmoamly, Stavroula Chante, Sumaiyyah Gauhar, Syed Ashraf, Tabinda Kharodia and Zhane Peterkin; University Hospitals of Leicester NHS Trust: Isgro Graziella and Hakeem Yusuff; University Hospitals of North Midlands NHS Trust: David Sutton, Ian Massey, Jade Di-Silvestro, Joanne Hiden, Mia Johnson and Richard Buka; University Hospitals Plymouth NHS Trust: Claire Lentaigne, Jackie Wooding and Nicola Crosbie; Whittington Health NHS Trust: Ana Alvaro, Emma Drasar, Elen Roblin, Georgina Santiapillai, Kathryn Simpson, Kayleigh Gilbert, Yanrong Jiang, Zara Sayar and Zehraa Al-Khafaji.
