# Supplementary material for: Autoimmune disease and COVID-19: a multicentre observational study in the United Kingdom
Source: Rheumatology (Oxford). 2022 Apr 4;61(12):4643–55. doi: 10.1093/rheumatology/keac209 (PMC8992350; doi:10.1093/rheumatology/keac209)

**Supplementary appendix:** **Autoimmune disease and COVID-19- a multicentre observational study in the United Kingdom**

**Table of contents**

**Study participating centres 1**

## **Table S1. International society on thrombosis and haemostasis criteria for major and clinically relevant non-major bleeding 2**

**Table S2: Type of autoimmune disease included in the study group for patients with autoimmune disease 3**

**Table S3. Details of the immunosuppressive treatment in patients with autoimmune disease prior to admission with COVID-19 4**

**Figure S1: Propensity matching for demographics and comorbidities in patients with autoimmune disease and no autoimmune disease 5**

# **Study Participating Centres**

1.Aberdeen Royal Infirmary

2.Aneurin Bevan University Health Board

3.Buckinghamshire Healthcare NHS Trust

4.Cardiff Haemophilia Centre

5.County Durham and Darlington NHS Foundation Trust

6.Cwm Taf Morgannwg University Health Board

7.Hampshire Hospitals NHS Foundation Trust

8.Imperial College Healthcare NHS Trust

9.King's College Hospital NHS Foundation Trust

10.Newcastle Hospitals NHS Foundation Trust

11.North Cumbria Integrated Care NHS FT

12.North Tees and Hartlepool NHS Foundation Trust

13.Oxford Haemophilia and Thrombosis Centre

14. Royal Brompton and Harefield Hospitals

15.Royal Free London NHS Foundation Trust

16. Royal Liverpool and Broadgreen University Hospitals NHS Trust

17. The Royal London Hospital - Barts Health NHS Trust

18. Royal Papworth Hospital NHS Foundation Trust

19. Sheffield Teaching Hospitals NHS Foundation Trust

20. South Tees NHS Foundation Trust

21. University Hospitals Birmingham NHS Foundation Trust

22. University Hospitals of Leicester NHS Trust

23. University Hospitals of the North Midlands

24. University Hospitals Plymouth NHS Trust

25. Whittington Health NHS Trust

26. Northumbria Healthcare Trust

| Major bleeding |
| --- |
| 1.Fatal bleeding, and/or  2.Bleeding in a critical area or organ, such as intracranial, intraspinal, intraocular, retroperitoneal, intra‐articular or pericardial, or intramuscular with compartment syndrome, and/or  3.Bleeding causing a fall in haemoglobin level of ≥ 20 g/ L or leading to transfusion of two or more units of whole blood or red cells. |
| Clinically relevant non‐major bleeding |
| 1.requiring medical intervention by a healthcare professional  2. leading to hospitalization or increased level of care  3. prompting a face to face (i.e., not just a telephone or electronic communication) evaluation |

## **Table S1. International society on thrombosis and haemostasis criteria for major and clinically relevant non-major bleeding**

| **Type of autoimmune disease** | **Number (%) (total =394)** |
| --- | --- |
| Antiphospholipid syndrome | 9 (2.3%) |
| ankylosing spondylitis | 30 (7.6%) |
| Autoimmune hepatitis | 25 (6.3%) |
| bullous pemphigoid | 6 (1.5%) |
| Coeliac Disease | 35 (8.9%) |
| Immune thrombocytopenic purpura | 32 (8.1%) |
| Mixed connective tissue disease | 36 (9.1%) |
| Polymyalgia rheumatica | 66 (16.6%) |
| rheumatoid arthritis | 37 (9.3%) |
| sarcoidosis | 15 (3.8%) |
| Seronegative Inflammatory Arthropathy | 48 (12.2%) |
| Sjogren's syndrome | 21 (5.3%) |
| systemic lupus erythematosus | 34 (8.6%) |

**Table S2: Type of autoimmune disease included in the study group for patients with autoimmune disease.** Antiphospholipid syndrome, systemic lupus erythematosus and rheumatoid arthritis are classified as severe rheumatologic autoimmune disease.

**Table S3. Details of the immunosuppressive treatment in patients with autoimmune disease prior to admission with COVID-19**

| **Immunosuppressive Treatment** | **SLE (n=34)** | **APS (n=9)** | **Rheumatoid arthritis (n=37)** | **Other autoimmune diseases** |
| --- | --- | --- | --- | --- |
| Steroids | 15/34 (44.2%) | - | 10/37 (27.0%) | 53/314 (16.8%) |
| Previous treatment with Rituximab in the last 12 months | 9/34 (26.5%) | - | 7/37 (18.9%) | - |
| Methotrexate, | - | - | 12/37 | - |
| Other immunosuppressive medications* | 29/34 (85.3%) | 3/9** (33.5%) | 2/37 (11.1%) | 10/314 (3.2%) |

*Include cyclosporin, azathioprine, mycophenolate mofetil and cyclosporine, hydroxychloroquine

** hydroxychloroquine

APS = antiphospholipid syndrome; SLE = systemic lupus erythematosus

**Figure S1: Propensity matching for demographics and comorbidities in patients with autoimmune disease and no autoimmune disease**


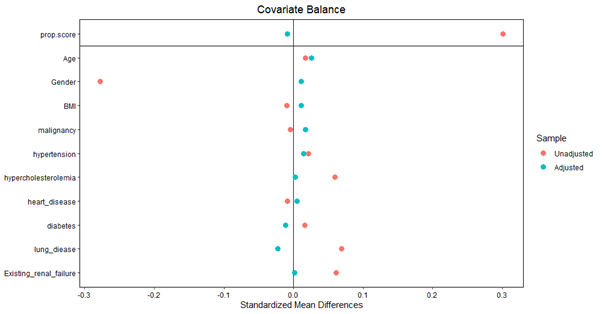

Supplement: keac209_Supplementary_Data [file keac209_supplementary_data.docx]
